# Supplementary material for: Mutations in PCYT2 disrupt etherlipid biosynthesis and cause a complex hereditary spastic paraplegia
Source: Brain. 2019 Oct 22;142(11):3382–97. doi: 10.1093/brain/awz291 (PMC6821184; doi:10.1093/brain/awz291)
Supplement: awz291_Supplementary_Data [file awz291_supplementary_data.zip › awz291-Suppl_data/Supplementary_Data4.zip!/Supplementary information 4 - plasma lipidomics.zip!/Plasma lipidomics/Control vs Patient/Statistical plots and data/LPC species/Heatmap.pdf]

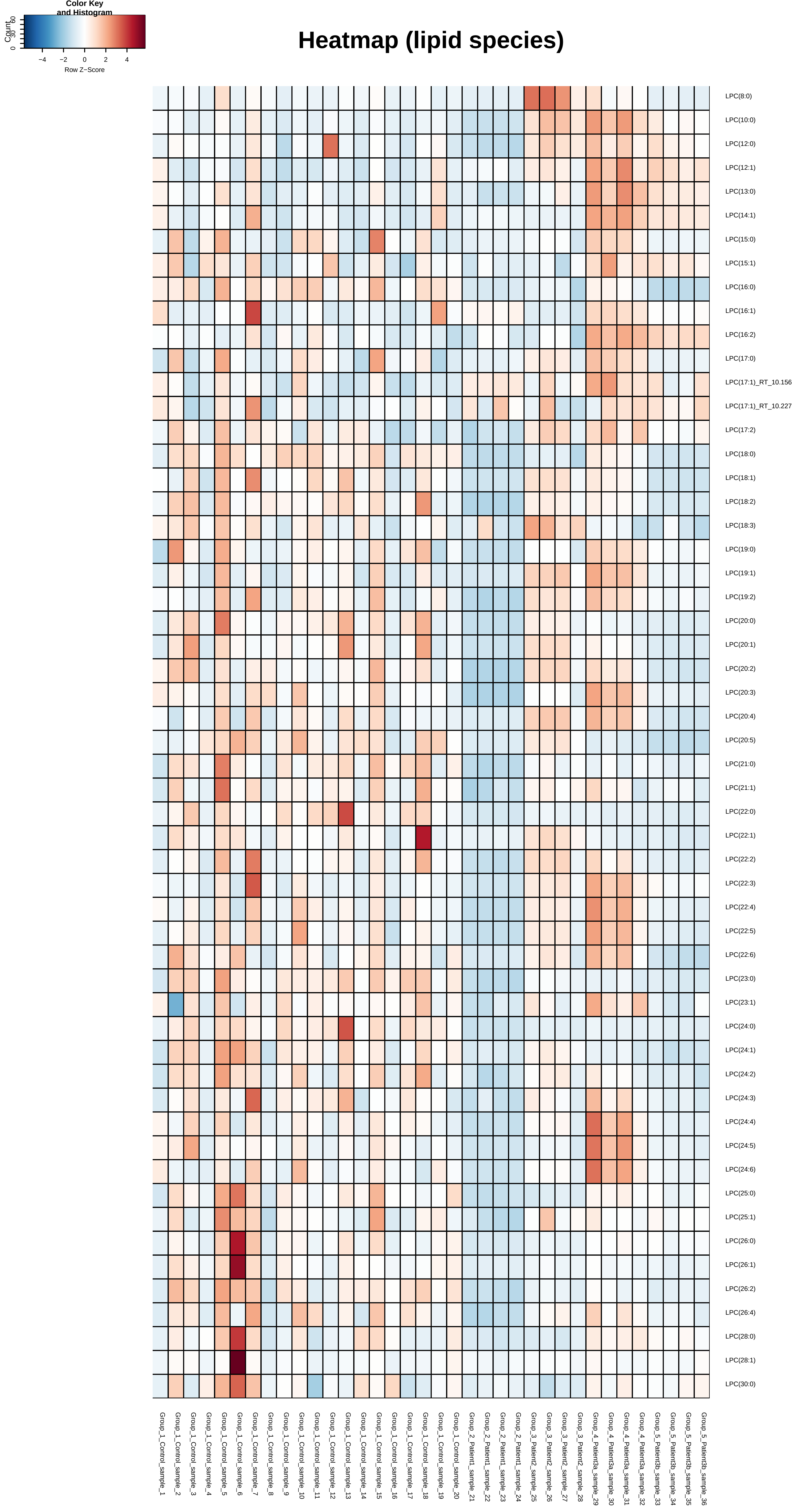
Color Key
and Histogram
Count
0
30
60
−4
−2
0
2
4
Row Z−Score
Heatmap (lipid species)
LPC(8:0)
LPC(10:0)
LPC(12:0)
LPC(12:1)
LPC(13:0)
LPC(14:1)
LPC(15:0)
LPC(15:1)
LPC(16:0)
LPC(16:1)
LPC(16:2)
LPC(17:0)
LPC(17:1)_RT_10.156
LPC(17:1)_RT_10.227
LPC(17:2)
LPC(18:0)
LPC(18:1)
LPC(18:2)
LPC(18:3)
LPC(19:0)
LPC(19:1)
LPC(19:2)
LPC(20:0)
LPC(20:1)
LPC(20:2)
LPC(20:3)
LPC(20:4)
LPC(20:5)
LPC(21:0)
LPC(21:1)
LPC(22:0)
LPC(22:1)
LPC(22:2)
LPC(22:3)
LPC(22:4)
LPC(22:5)
LPC(22:6)
LPC(23:0)
LPC(23:1)
LPC(24:0)
LPC(24:1)
LPC(24:2)
LPC(24:3)
LPC(24:4)
LPC(24:5)
LPC(24:6)
LPC(25:0)
LPC(25:1)
LPC(26:0)
LPC(26:1)
LPC(26:2)
LPC(26:4)
LPC(28:0)
LPC(28:1)
LPC(30:0)
Group_1_Control_sample_1
Group_1_Control_sample_2
Group_1_Control_sample_3
Group_1_Control_sample_4
Group_1_Control_sample_5
Group_1_Control_sample_6
Group_1_Control_sample_7
Group_1_Control_sample_8
Group_1_Control_sample_9
Group_1_Control_sample_10
Group_1_Control_sample_11
Group_1_Control_sample_12
Group_1_Control_sample_13
Group_1_Control_sample_14
Group_1_Control_sample_15
Group_1_Control_sample_16
Group_1_Control_sample_17
Group_1_Control_sample_18
Group_1_Control_sample_19
Group_1_Control_sample_20
Group_2_Patient1_sample_21
Group_2_Patient1_sample_22
Group_2_Patient1_sample_23
Group_2_Patient1_sample_24
Group_3_Patient2_sample_25
Group_3_Patient2_sample_26
Group_3_Patient2_sample_27
Group_3_Patient2_sample_28
Group_4_Patient3a_sample_29
Group_4_Patient3a_sample_30
Group_4_Patient3a_sample_31
Group_4_Patient3a_sample_32
Group_5_Patient3b_sample_33
Group_5_Patient3b_sample_34
Group_5_Patient3b_sample_35
Group_5_Patient3b_sample_36
